# Spinal cord injury modulates the lung inflammatory response in mechanically ventilated rats: a comparative animal study

**DOI:** 10.14814/phy2.13009

**Published:** 2016-12-30

**Authors:** Karine Truflandier, Eric Beaumont, Karim Maghni, Michel De Marchie, Emmanuel Charbonney, Jadranka Spahija

**Affiliations:** ^1^ Research CenterSacré‐Cœur Hospital of MontrealDepartment of MedicineUniversité de MontréalMontrealQuebecCanada; ^2^ Department of Biomedical SciencesQuillen College of MedicineEast Tennessee State UniversityJohnson CityTennessee; ^3^ Department of Adult Critical CareJewish General HospitalMcGill UniversityMontréalQuebecCanada; ^4^ School of Physical and Occupational TherapyMcGill UniversityMontrealQuebecCanada; ^5^ Center for Interdisciplinary Research in Rehabilitation in MontrealJewish Rehabilitation HospitalLavalQuebecCanada

**Keywords:** Mechanical ventilation, oxidative stress response, pulmonary inflammation, spinal cord injury

## Abstract

Mechanical ventilation (MV) is widely used in spinal injury patients to compensate for respiratory muscle failure. MV is known to induce lung inflammation, while spinal cord injury (SCI) is known to contribute to local inflammatory response. Interaction between MV and SCI was evaluated in order to assess the impact it may have on the pulmonary inflammatory profile. Sprague Dawley rats were anesthetized for 24 h and randomized to receive either MV or not. The MV group included C4–C5 SCI, T10 SCI and uninjured animals. The nonventilated (NV) group included T10 SCI and uninjured animals. Inflammatory cytokine profile, inflammation related to the SCI level, and oxidative stress mediators were measured in the bronchoalveolar lavage (BAL). The cytokine profile in BAL of MV animals showed increased levels of TNF‐*α*, IL‐1*β*, IL‐6 and a decrease in IL‐10 (*P* = 0.007) compared to the NV group. SCI did not modify IL‐6 and IL‐10 levels either in the MV or the NV groups, but cervical injury induced a decrease in IL‐1*β* levels in MV animals. Cervical injury also reduced MV‐induced pulmonary oxidative stress responses by decreasing isoprostane levels while increasing heme oxygenase‐1 level. The thoracic SCI in NV animals increased M‐CSF expression and promoted antioxidant pulmonary responses with low isoprostane and high heme oxygenase‐1 levels. SCI shows a positive impact on MV‐induced pulmonary inflammation, modulating specific lung immune and oxidative stress responses. Inflammation induced by MV and SCI interact closely and may have strong clinical implications since effective treatment of ventilated SCI patients may amplify pulmonary biotrauma.

## Introduction

Spinal cord injury (SCI) has serious consequences on morbidity and mortality (Lee et al. 2014). Respiratory muscle weakness and paralysis, especially following cervical SCI, can lead to respiratory insufficiency and a need for mechanical ventilatory support. However, there is accumulated evidence that mechanical ventilation (MV) can contribute to the development of ventilator‐induced lung injury (VILI) leading to pulmonary inflammation (Slutsky and Ranieri 2013), endothelial and epithelial dysfunction, alteration of surfactant production, and lung edema augmenting damage in previously injured lungs (Chiumello et al. 1999; Ranieri et al. 1999). Prolonged MV, using conventional tidal volumes (*V*_T_) or airway pressures, can also lead to biotrauma characterized by a release of inflammatory cytokines (Vaneker et al. 2007; Determann et al. 2010), even when applied to healthy lungs. Both clinical and experimental studies have reported that MV induces lung neutrophilia and increased levels of interleukin IL‐1*β* and IL‐6 and tumor necrosis factor‐*α* (TNF‐*α*), among the other chemokines (Bailey et al. 2008; Syrkina et al. 2008). Cyclic stretching of lung tissue (Mourgeon et al. 2000) has been shown to result in an increased cytokine production associated with epithelial cell (Vlahakis et al. 1999) and alveolar macrophage activity (Wu et al. 2013). Since higher levels of proinflammatory cytokines IL‐6 and TNF‐*α* have been reported in ventilated animals in contrast to higher levels of anti‐inflammatory cytokine IL‐10 in nonventilated animals (Tremblay et al. 1997), we may expect specific patterns of cytokine expression induced by MV.

Alveolar macrophages are the main cellular source of cytokines for pulmonary defense. Macrophages can exhibit different and specific phenotypes for cytokine and chemokine expression during immune infection, tissue development, and repair (Mosser and Edwards 2008). Macrophage populations have been classified into different subtypes based on their phenotypes, referred to as macrophage polarization: (1) classical activation (M1) and (2) alternative activation (M2). M1 cells are known to be driven by granulocyte macrophage colony‐stimulating factor (GM‐CSF) and induced or primed by interferon‐*γ* (IFN‐*γ*) and lipopolysaccharide (LPS) (Mantovani et al. 2007). In contrast, M2 are driven by macrophage colony‐stimulating factor (M‐CSF) as well as induced by IL‐4, IL‐13, or IL‐21 (Sica and Mantovani 2012). M1 mainly releases IL‐1*β*, IL‐6, TNF‐*α*, and the macrophage inflammatory protein‐1 alpha (MIP‐1*α*) (Martinez et al. 2008), whereas M2 mainly produces IL‐10 and monocyte chemotactic protein‐1 (MCP‐1) (Gordon and Martinez 2010). M2 cells have been recognized for their anti‐inflammatory properties.

The central nervous system plays a significant role in regulating immunity both in humans and animals (Meisel et al. 2005). SCI has been shown to trigger local (Fleming et al. 2006) and systemic inflammatory responses which may affect distal organs. In nonventilated rats, SCI induces neutrophil infiltrations, increases phagocytic macrophages, proinflammatory enzymatic activity (e.g., myeloperoxidase and matrix metalloproteinase‐9), and oxidative stress responses (e.g., lipid peroxidation) in the lungs (Gris et al. 2008). Ventilation can also trigger the pulmonary immune system reactivity even in the absence of prior lung inflammation (Altemeier et al. 2004). However, whether or not interplay between inflammatory responses to SCI and MV may amplify or reduce biotrauma and lung injury development is currently unknown.

Here, in an animal model, our data provides the first evidence that mechanical ventilation at low tidal volume can interact with the inflammatory pulmonary response, and that SCI modulates the innate immune system by selectively decreasing pulmonary IL‐1*β* expression. We further demonstrate that SCI has no effect on mechanical ventilation‐induced lung neutrophilia, but markedly and selectively decreases oxidative stress responses. Our study reveals an unexpected role of SCI and highlights the modulation of the pulmonary inflammation induced by MV in the presence of SCI.

## Material and Methods

### Study design

The present study was designed to test the hypothesis that spinal cord injury influences mechanical ventilation‐induced lung inflammation. We measured cell counts, leukocytes types, classic inflammatory mediators, cell injury markers, macrophage phenotype markers, and oxidative stress.

### Study approval

All procedures were conducted according to the recommendations of the Canadian Council for Animal Care and were approved by the Animal Ethics Committee of the Research Center of Sacré‐Coeur Hospital of Montreal.

### Animal preparation

Thirty‐three adult female Sprague Dawley rats (225–250 g, Charles River, St‐Constant, Quebec, Canada) were used in our study. Rats were randomized into five separate groups: three groups received MV, whereas two others did not. For ventilated rats, one group received a cervical SCI (*N* = 7, C4–C5), one group a thoracic SCI (*N* = 7, T10), and the last group was uninjured, that is, no SCI (*N* = 7). For the rats without MV, one group received a thoracic SCI (*N* = 6, T10), while the other was uninjured, that is, no SCI (*N* = 6). A nonventilated group of cervical SCI could not be included given the risk of respiratory insufficiency. All rats were initially anesthetized with ketamine and xylazine (90/10 mg/kg) administered intramuscularly. Rats were administered these two agents through a catheter placed in the jugular vein, with dosage adjusted to 0.9 mL/h for 24 h. All animals were placed on a heating blanket for the duration of the experiment to prevent a drop in body temperature. Body temperature was continuously monitored and maintained between 37°C and 39°C. Heart rate was monitored at the paw, using subcutaneous electrodes (Nihon Kohden, Tokyo, Japan). All animals received the following procedures every 4 h: (1) mobilization of the extremities to compensate for the loss of venous return, (2) manual massaging of the bladder to prevent renal stasis.

### Mechanical ventilation

In the intubated ventilated animals, the ventilator support was set before each laminectomy (SCI or sham). The ventilator settings (Kent Scientific, Topo, Torrington, CT) were maintained for 24 h to deliver an appropriate tidal volume (6 mL/kg) based on the animal's body weight according to the nomogram of Kleinman and Radford (1964), with constant (1) peak inspiratory airway pressure (PIP) 10 cm H_2_O, (2) respiratory rate (RR) 60 breaths per minute, (3) inspiration‐to‐expiration ratio of 1:3 with inspiration setting at 35%, and (4) expired end‐tidal CO_2_ between 2.5% and 3.6% (Capstar‐100 CO_2_, analyzer). For nonventilated animals, supplemental oxygen was given (0.1 L/min).

### Spinal cord injury

A laminectomy was performed at C4 or T9 vertebrae, exposing the C5 or T10 spinal cord segment. SCI was performed as described previously (Dery et al. 2009). Briefly, the stereotactic clamps were installed on both sides of the laminectomy to stabilize the spine of injured animals prior to injury. To produce a moderate SCI, a weight of 5 g was dropped from a 6‐cm height directly on the spinal cord (30 g/cm impact). For uninjured animals, all other surgical procedures were performed except for the laminectomy or the impact to the spinal cord. Anesthetized rats were euthanized by exsanguination 24 h postinjury.

### Bronchoalveolar lavage

In order to proceed to a bronchoalveolar lavage (BAL), the NV animals received a tracheotomy (endotracheal tube secured in place using surgical thread). The right lung was then clamped at the bronchus level and the left lung was subjected to five consecutive lavages through the tracheal tube as described previously (Maghni et al. 2000). Each BAL was performed by introducing 2.5 mL of filtered sterile phosphate‐buffered saline (PBS) containing 0.5% of bovine serum albumin (2.5 mL PBS/BSA) into the tube extremity and drawn up by a 5‐mL syringe placed at the other extremity. The fluid recovered from the first wash was injected into a 15‐mL falcon tube, while the remaining four subsequent washes where transferred to a second separate falcon tube. Both tubes were placed on ice in order to keep cells alive and to be processed for measurement of total cell counts, inflammatory mediators, and oxidative stress status. The material obtained from the first wash was centrifuged at 350 g for 10 min at 4°C and the supernatant was harvested and aliquoted in Eppendorf tubes kept at −80°C until assessment of mediator analysis. Percentages of neutrophils, macrophages, and lymphocytes were determined from the remaining pellet. The cell pellet was resuspended in 1 mL of BAL fluid provided from the second series of lung washes and the cell suspension was centrifuged once again. Cells were counted with a hemacytometer and viability was assessed by the trypan blue dye exclusion test. The differential cell counts were determined on a cytospin slide that was prepared with a Cytospin model II (Shandon, Pittsburg, PA) and slides stained with Accustain‐modified Giemsa for differential cell counts.

### Determination of cellular phenotype factors and markers

Concentrations of cytokines and chemokines (IL‐6, IL‐10, IL‐1*β*, and TNF‐*α*) were determined from the BAL fluid and normalized based on a protein assay by standard Bradford assay method (Dery et al. 2009). Markers for M1 and M2 phenotypes were determined using a commercial Bio‐Plex cytokine assay based on the Luminex technology (Bio‐Rad Laboratories, Inc., Mississauga, Ontario, Canada) as described previously (Malloy et al. 1997). A standard curve was performed to the ELISA test for the immunoassay procedure and used for the standardization of the signal.

Specific M1 phenotype factors were profiled by detection of granulocyte macrophage colony‐stimulating factor (GM‐CSF) and by detection of phenotype markers which were determined with macrophage inflammatory protein‐1 (MIP‐1*α*), interleukin 12p70 (IL‐12p70), and interferon‐inducible protein 10 (IP‐10). Monocyte chemotactic protein‐1 (MCP‐1) and macrophage colony‐stimulating factor (M‐CSF) were determined as cellular markers specific to M2 phenotype and were quantified in BAL fluids using an M‐CSF ELISA kit according to the manufacturer's instructions (NovaTeinbio, Inc., Cambridge, MA).

### Determination of lung injury markers

Alkaline phosphatase (AP), as a reflection of type II epithelial cell necrosis, and lactate dehydrogenase (LDH) were assessed in BAL fluids as markers of lung injury as described previously (Noel et al. 2012). Briefly, levels of AP were quantified using a QuantiChrom^™^ Alkaline Phosphatase Assay kit (Gentaur, Genprice, Inc., Santa Clara, CA). Levels of LDH were assessed using the cytotoxicity detection kit^plus^ from Roche Diagnostics GmbH (Roche Applied Science, Mannheim, Germany).

### Determination of oxidative stress responses

Levels of 8‐isoprostane in BAL fluid were determined using the 8‐iso prostaglandin F2 immunoassay kit (Cayman Chemical Company, Ann Arbor, MI) as previously described (Noel et al. 2012). Intracellular levels of heme oxygenase‐1 (HO‐1) in total BAL cell extracts were quantified using the rat ImmunoSet^Tm^ H0‐1 ELISA development kit (Enzo Life Sciences, Inc., Farmingdale NY). Total BAL cell extracts were obtained by lysing cells in the Cell Lytic solution (Sigma Aldrich, Millipore Corporation, Billerica, MA) containing a cocktail of protease inhibitors (Sigma Aldrich, Millipore Corporation).

### Statistical analysis

Statistical analyses were performed using PASW Statistics 18 (SPSS, Inc., Chicago, IL). Comparisons of mean values of pulmonary cell counts, and inflammatory and oxidative stress mediators for the three mechanically ventilated groups were made using a one‐way analysis of variance (ANOVA) followed by Tukey's post hoc test, to look at the effect of SCI level on inflammatory responses in the lungs.

The effect of MV and presence of a lesion (thoracic) were evaluated using a two‐way ANOVA over four groups (MV‐NL, MV‐TL, NV‐NL, and NV‐TL) followed by unpaired *t*‐tests for post hoc contrasts of significant effects. Statistical differences were considered significant at *P *<* *0.05. All data are presented as means ± standard error of means (SEM).

## Results

### Vital parameters in mechanically ventilated (MV) and nonventilated (NV) animals

Mean values of vital parameters for all groups are summarized in Table 1.

**Table 1:** Summary of vital parameters in mechanically ventilated and nonventilated rats

	Groups				
	MV‐NL	MV‐CL	MV‐TL	NV‐NL	NV‐TL
CO_2_ Exp (%)	3.0 (0.3)	2.9 (0.3)	3.6 (0.5)	–	–
Peak insp pressure (cm H_2_O)	11.1 (0.1)	11.6 (0.3)	10.9 (0.2)	–	–
Respiratory rate (breaths/min)	63.1 (1.4)	62.9 (1.0)	61.0 (0.4)	72.9 (6.8)	74.5 (4.9)
Heart rate (bpm)	248.2 (17.8)	239.6 (13.0)	229.1 (9.5)	248.0 (9.6)	224.5 (6.0)
Temperature (°C)	36.8 (0.1)	36.8 (0.1)	36.8 (0.1)	36.6 (0.1)	36.8 (0.2)

Values are mean (±SEM) of individual rats per group. Exp, expiratory; Insp: inspiratory; MV, mechanical ventilation; NL, no lesion; CL, cervical lesion; TL, thoracic lesion; NV, no ventilation. Comparisons of means for the three ventilated groups were made using a one‐way ANOVA followed by Tukey's post hoc test and a two‐way ANOVA followed by unpaired *t*‐tests for post hoc contrasts of significant effects evaluated MV and thoracic lesion effect. *P *<* *0.05.

### Effects of SCI on MV‐induced BAL neutrophilia and lung damage

Mechanical ventilation resulted in significantly higher neutrophil levels and total cell counts in BAL, but did not affect the number of alveolar macrophages (Table 2). Alkaline phosphatase (AP) activity levels in the BAL fluids were significantly higher in all MV groups compared to respective controls (Table 2). There was no significant change in LDH levels in BAL fluid among all groups of animals (Table 2). Having a SCI did not modify cell counts or AP levels within the respective groups of MV or NV.

**Table 2:** BAL total and differential cell counts and BAL markers of lung cell damage

	Groups							
	MV‐NL	MV‐CL	MV‐TL	*P* one‐way ANOVA (lesion)	NV‐NL	NV‐TL	*P* two‐way ANOVA (lesion)	*P* two‐way ANOVA (MV)
Total	3.7 (0.7)	5.2 (0.9)	4.2 (0.6)	0.400	2.0 (0.3)	2.6 (0.6)	0.331	0.011
Macro	1.5 (0.4)	3.0 (0.6)	1.8 (0.5)	0.172	1.7 (0.3)	1.9 (0.3)	0.505	0.749
Neutro	2.5 (0.4)	2.6 (0.4)	3.4 (0.1)	0.926	0.1 (0.0)*	0.2 (0.2)‡	0.982	<0.001
Lympho	0.0 (0.0)	0.0 (0.0)	0.1 (0.0)	0.345	0.1 (0.0)	0.3 (0.2)	0.322	0.123
AP (pg/ml)	36.0 (9.3)	22.6 (3.6)	38.8 (7.0)	0.293	8.2 (1.3)*	10.2 (0.6)†	0.720	<0.001
LDH (au)	3.1 (2.7)	0.3 (0.2)	0.6 (0.4)	0.432	5.5 (3.5)	0.1 (0.0)	0.081	0.656

Mean values (±SEM) of individual rats per group. Total and differential cell counts are expressed as millions of cells. MV, mechanical ventilation; NL, no lesion; CL, cervical lesion; TL, thoracic lesion; NV, no ventilation. Macro, macrophages; Neutro, neutrophils; Lympho, lymphocytes; AP, alkaline phosphatase; LDH, lactate dehydrogenase; au, arbitrary units.

*P <* *0.05 for post hoc contrasts versus MV‐NL.

^†^ *P *<* *0.05 and ^‡^ *P* < 0.001 for post hoc contrasts versus MV‐TL.

Presence of a cervical lesion (CL) or alternatively a thoracic lesion (TL) in MV animals had no differential effect on cell counts or biochemical markers (Table 2).

### Effects of MV and SCI on BAL cytokines expression

**Figure 1:**
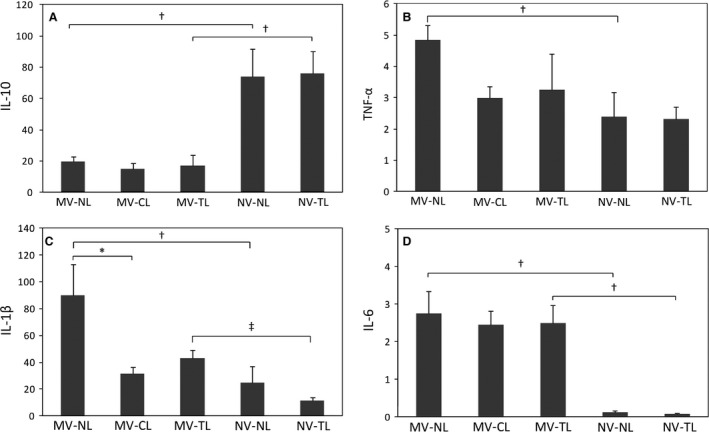
Effects of MV and SCI on total BAL cytokine expression. Mean values (±SEM). (A) Interleukin‐10 in pg/mL, (B) TNF‐*α* in pg/mL, (C) interleukin‐1*β* in pg/mL, (D) interleukin‐6 in μg/mL; MV, mechanical ventilation; NL, no lesion; CL, cervical lesion; TL, thoracic lesion; NV, no ventilation. *P < 0.05 for post hoc contrasts (one‐way ANOVA). ^†^ *P* < 0.05 and ^‡^ *P* < 0.001 for post hoc contrasts (two‐way ANOVA).

Mechanical ventilation had a significant effect on the cytokine profile (Fig. 1) (*P* two‐way ANOVA: TNF‐*α*,* P* = 0.043; IL‐1*β*,* P* = 0.003; IL‐6, *P* < 0.001; IL‐10, *P* < 0.001). Post hoc contrasts revealed a significant increase of TNF‐*α* (*P* = 0.018), IL‐1*β* (*P* = 0.038), IL‐6 (*P* < 0.001), and a decrease in IL‐10 (*P* = 0.007) with MV in the absence of a SCI (MV‐NL vs. NV‐NL). Presence of SCI did not modify the levels of IL‐6 and IL‐10 in the MV or NV groups. The presence of a CL in MV animals significantly reduced IL‐1*β* level in BAL (*P* = 0.030), whereas the decrease with a TL did not reach statistical significance (*P* = 0.075) (Fig. 1).

### Effects of MV and SCI on BAL markers related to macrophages types and injured cells

The analysis of mediators previously identified in in vitro studies as specific markers for M1 and M2 macrophages were classified by phenotype groups (Table 3) (Martinez et al. 2009). MIP‐1*α* levels, a chemokine previously identified as a marker when epithelial cells are injured (Martinez et al. 2009), was higher in the BAL of MV animals (*P* < 0.001) compared to their respective controls. MIP‐1*α* levels were not altered by SCI or by the level of the lesion. MV and SCI had no effect on the levels of GM‐CSF, IL‐12p70, and IP‐10 (Table 3).

**Table 3:** BAL cytokines and chemokines related to specific injured cell

	Groups							
	MV‐NL	MV‐CL	MV‐TL	*P* one‐way ANOVA (lesion)	NV‐NL	NV‐TL	*P* two‐way ANOVA (lesion)	*P* two‐way ANOVA (MV)
M1 phenotype								
GM‐CSF	1.2 (0.9)	5.3 (3.3)	0.5 (0.5)	0.133	1.2 (1.2)	2.9 (1.8)	0.659	0.303
MIP‐1*α*	136.1 (12.9)	110.7 (17.2)	114.5 (18.9)	0.514	0.0 (0.0)†	27.1 (20.5)‡	0.863	<0.001
IL‐12p70	3.8 (1.8)	0.7 (0.5)	7.1 (2.3)	0.098	5.2 (2.2)	4.3 (1.2)	0.545	0.710
IP‐10	0.0 (0.0)	3.9 (3.9)	1.0 (1.0)	0.373	0.0 (0.0)	0.0 (0.0)	0.368	0.368
M2 phenotype								
M‐CSF	2.7 (1.8)	8.0 (7.9)	13.7 (8.4)	0.516	6.3 (1.8)	25.7 (2.8)‖	0.009	0.160
MCP‐1	498.4 (115.8)	202.5 (48.7)	420.6 (82.0)	0.126	5.8 (4.5)*	11.6 (8.7)§	0.646	<0.001

Mean values (±SEM). BAL cytokines and chemokines are expressed as pg/mL. MV, mechanical ventilation; NL, no lesion; CL, cervical lesion; TL, thoracic lesion; NV, no ventilation; GM‐CSF, granulocyte macrophage colony‐stimulating factor; M‐CSF, macrophage colony‐stimulating factor; MIP‐1*α*, macrophage inflammatory protein‐1; IL‐12p70, interleukin‐12p70; MCP‐1, monocyte chemotactic protein‐1; IP‐10, interferon gamma‐induced protein 10.

*P <* *0.05 and ^†^ *P *<* *0.001 for post hoc contrasts versus MV‐NL.

^‡^ *P *<* *0.05 and ^§^ *P *<* *0.001 for post hoc contrasts versus MV‐TL.

^‖^ *P *<* *0.001 for post hoc contrasts versus NV‐NL.

For markers related to the M2 phenotype, M‐CSF levels were higher (*P* = 0.009) in the group with TL compared to NL, but only in the NV animals (Table 3). Next, we assessed the level of MCP‐1, a chemokine also previously identified as a specific marker of injured epithelial cell, in the BAL fluid. MCP‐1 levels were significantly higher (*P* < 0.001) in the MV groups, whereas the presence of SCI had no modifying effect. CL or TL did not show significant differences (Table 3).

### MV and SCI‐induced oxidative stress responses

**Figure 2:**
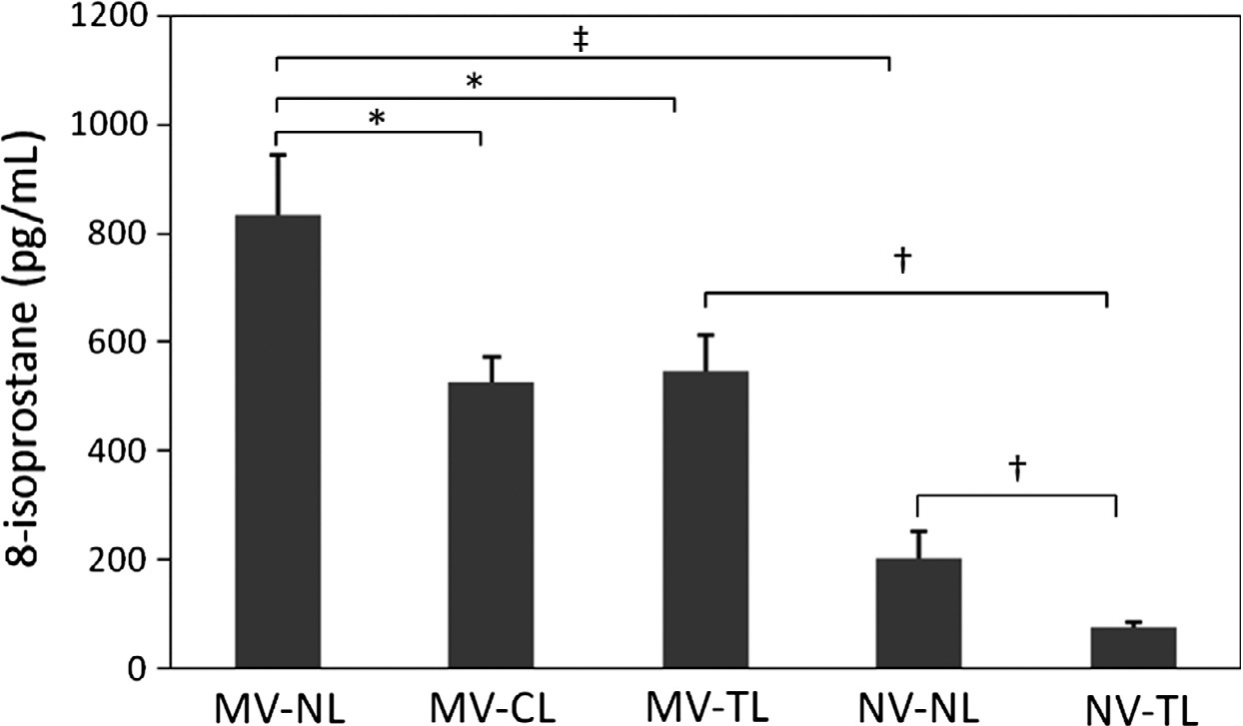
MV‐induced oxidative stress responses. Mean values (±SEM) of 8‐isoprostane in pg/mL; MV, mechanical ventilation; NL, no lesion; CL, cervical lesion; TL, thoracic lesion; NV, no ventilation. *P < 0.05 for post hoc contrasts (one‐way ANOVA). ^†^ *P* < 0.05 and ^‡^ *P* < 0.001 for post hoc contrasts (two‐way ANOVA).

Bronchoalveolar lavage fluid levels of 8‐isoprostane, a marker of lipid peroxidation in response to oxidative burst (Fritsch‐Decker et al. 2011), were first examined to evaluate the oxidative stress response. As illustrated in Figure 2, the level of 8‐isoprostane was significantly higher in the MV animals without SCI compared to NV. However, significantly lower levels of 8‐isoprostane were observed in the presence of SCI both in MV and NV animals (Fig. 2). The 8‐isoprostane level was hampered as much by the CL (*P* = 0.050) as the TL (*P* = 0.048) in the MV group.

**Figure 3:**
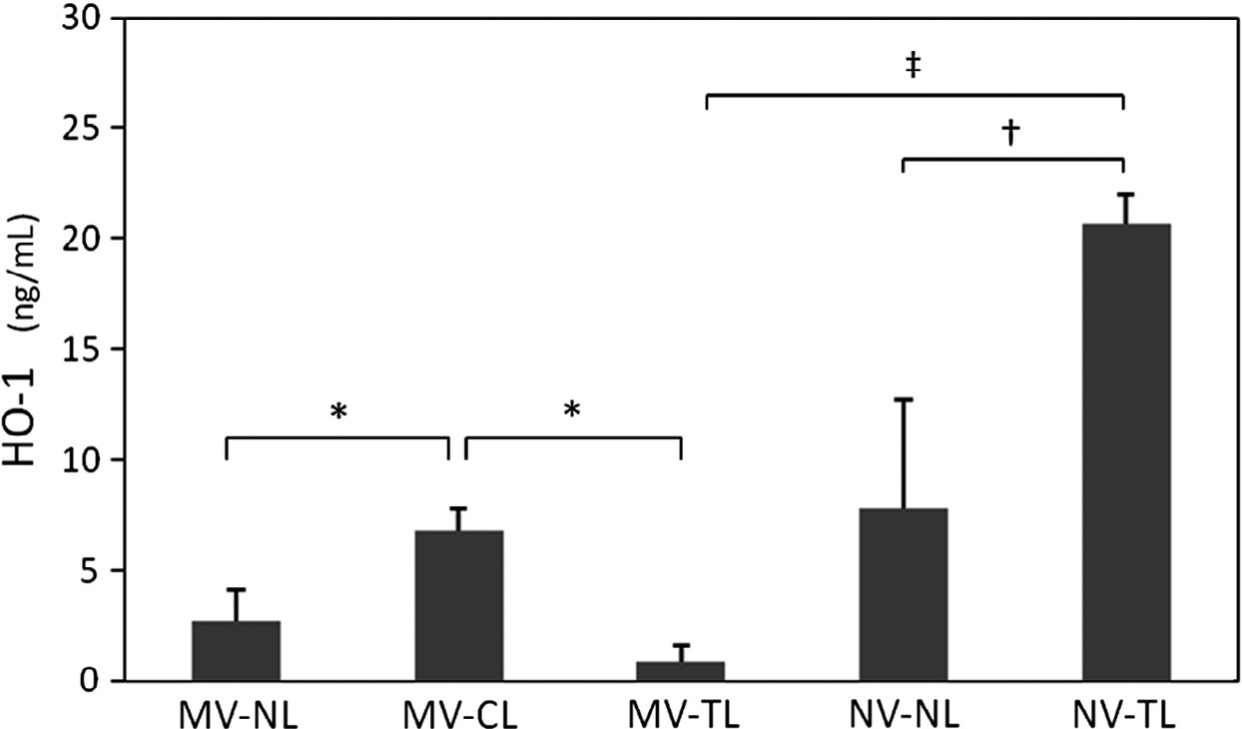
Heme oxygenase‐1 in pulmonary cell extracts. Mean values (±SEM) of HO‐1 in ng/mL; MV: mechanical ventilation; NL, no lesion; CL, cervical lesion; TL, thoracic lesion; NV, no ventilation. *P < 0.05 for post hoc contrasts (one‐way ANOVA). ^†^ *P* < 0.05 and ^‡^ *P* < 0.001 for post hoc contrasts (two‐way ANOVA).

To further evaluate lung oxidative stress modulation in response to mechanical ventilation and SCI, we examined the expression of the heme oxygenase‐1 (HO‐1) protein, an intracellular enzyme involved in pulmonary defense by protecting lung cells and tissues against stress and injury (Ryter et al. 2006), in the pulmonary cellular extracts of total BAL cells. Interestingly, a TL in NV animals resulted in a significantly higher level of HO‐1 (*P* < 0.001) compared to no SCI. However, HO‐1 was not significantly different in MV animals with and without a TL. Finally, the CL animals in the MV group showed significant higher levels (*P* = 0.039 and *P* = 0.002) compared to those with NL and TL, respectively (Fig. 3).

## Discussion

In the context of spinal cord injury, MV is often part of the initial management and, if prolonged, contributes to the known morbidity of the patients (Jackson and Groomes 1994). After injury, in order to defend and repair the damaged tissues, the body implements a wide range of inflammatory responses, that is, cytokine production in various locations. To date, the inflammatory interplay between spinal cord injury and MV has not been investigated. Understanding the mechanism by which MV could modulate the cellular response to mechanical stress in spinal cord injured patients who need respiratory support is important clinically. Hence, it is interesting to determine if and how MV, after SCI, may affect the posttraumatic inflammation in the lungs. Although MV is applied in patients with respiratory failure, this respiratory support is known to induce pulmonary injury with massive neutrophil infiltration and elevated levels of proinflammatory cytokines IL‐1*β* and TNF‐*α* (Tremblay et al. 1997; Bailey et al. 2008). In clinical studies, conventional MV applied to patients with healthy lungs has been shown to increase proinflammatory cytokines that may contribute to the development of lung injury (Determann et al. 2010). Even in healthy lungs, repeated mechanical cyclic stretch of alveoli is an important induction factor of oxidative stress, which starts at the epithelial and endothelial levels, leading to an inflammatory cascade (Grembowicz et al. 1999; Vlahakis and Hubmayr 2003). In animal models, cytokine levels have been reported to increase progressively with duration of MV and then to reverse, once MV is stopped (Vaneker et al. 2007).

Similar to other reported animal models (Chiumello et al. 1999), the lungs of MV rats in the current study mainly showed an influx of neutrophils, increased levels of proinflammatory cytokines IL‐6, IL‐1*β*, TNF‐*α*, and a decreased IL‐10 level. Our results show that cervical SCI reduced the expression of IL‐1*β* assessed in BAL fluids of MV animals. LDH levels, which are a marker of cell influx or injury, were not significantly different, although there was a trend for reduction when SCI was present, in both MV and NV animals. In the literature, the activity of LDH measured in lungs is found to be highly dependent on the time point at which the material was obtained in relation to the last exposure to the causative antigen (Panus et al. 1989). It is therefore possible that the LDH levels we obtained were related to this time effect and different values might have been obtained if we measured at 8 or 12 h postinjury. Finally, our results show that the increased lipid peroxidation observed during MV was hampered by the presence of SCI.

Although the cytokines recovered in BAL fluids are not exclusively produced by the alveolar macrophages, they are key cells in the innate immune response in the lungs. They are also central for the resolution of inflammation and the parenchymal repair after injury. During MV, cyclic stretching of macrophages may also induce cytokine expression (Pugin et al. 1998). For that reason, we specifically addressed the expression of other selective markers of macrophage phenotypes. M1‐polarized macrophages are GM‐CSF driven (Mosser and Edwards 2008), and cells selectively produce IL‐12p70, IP‐10, and MIP‐1*α* (Mosser 2003). We found no increase in GMC‐CSF, IL‐12p70, and IP‐10 levels in MV‐NL animals when compared to NV‐NL. Levels of MIP‐1*α* were significantly higher in MV‐NL rats, while absent in NV‐NL animals. The absence of increased cytokines in our study suggests that MIP‐1*α* is probably not produced by alveolar macrophages but by other cells such as epithelial cells. M2‐polarized macrophages are M‐CSF driven (Mantovani et al. 2007), and cells preferentially release IL‐10, MCP‐1, and the antioxidative enzyme HO‐1 (Ryter et al. 2006). BAL fluid analysis revealed a basic state level of nonmechanically stressed cells as shown by the M‐CSF levels in NV‐NL animals that were not significantly different from the levels of M‐CSF recovered in BAL fluids of MV‐NL rats. In addition to IL‐6, TNF, and IL‐1*β*, MV also increased the expression of MCP‐1 in MV‐NL animals showing that MV affects the regulation of these cytokines. Cellular response to mechanical stretch of pulmonary endothelial cells and macrophages has been increasingly investigated and in support of our findings, previous studies (Dreyfuss et al. 2006; Altemeier et al. 2004) have shown that cyclic stretch induces the secretion of the chemokine MCP‐1, a well‐recognized mediator of lung inflammation and repair. Because of its potent monocyte chemotactic activity, MCP‐1 plays an essential role in recruiting and accumulating neutrophils to the lungs. MCP‐1 demonstrates significant upregulation when submitted to four hours of cyclic stretch as compared to cells grown in static conditions (Dreyfuss et al. 2006). Our in vivo model showed higher levels of MCP‐1 due to stretch even at low tidal volumes, as reported previously (Altemeier et al. 2004).

Subsequently, we examined whether SCI by itself or combined with MV could modulate influx of inflammatory cells in airways and impact lung damage. We first addressed the effect of lower thoracic lesion (T10 level) injury alone and found that animals having a TL showed no evidence of inflammation or lung injury and showed similar profiles of expression for IL‐10, IL‐1*β*, TNF, and IL‐6 cytokines when compared to NV‐NL rats. Interestingly, TL injury alone caused a significant increase in M‐CSF in BAL. This novel finding might suggest that thoracic SCI may have modulated the innate immune response in the lungs of nonventilated animals. Consequently, we examined whether a specific cytokine expression was induced when animals with TL were also ventilated. We found that TL modulated cytokine production, as illustrated by the decreased IL‐1*β* and IL‐10 levels but not IL‐6, in MV‐TL animals. However, there was no modulation of BAL neutrophils or lung injury in MV‐TL animals. We then examined whether the localization of the SCI could impact MV‐induced pulmonary inflammation. We found that the cervical (C4–C5 levels) and thoracic lesions in ventilated animals provoked a similar modulation of cytokine expression with no change in BAL neutrophils or the degree of lung injury. Taken together, our results support a new concept of the role of SCI for modulating specific immune responses, but not inflammatory cell influx or injury (LDH) in the lungs of MV animals. In nonventilated animals, SCI showed lower LDH levels in NV‐TL animals.

Spinal cord injury is well‐known to induce a strong local inflammatory response including a massive infiltration of neutrophils to the injured site (Fleming et al. 2006). To date, very few studies have looked at lungs of SCI rats (T4). According to Gris et al., neutrophils migrate directly from the blood stream to the lungs where they infiltrate the alveolar spaces 12 h postinjury. These neutrophils may increase proinflammatory enzymes (myeloperoxidases) and oxidative responses (Gris et al. 2008). However, few studies have also evaluated the effect of SCI level on immune function. One study observed increased neutrophils in the blood of animals with thoracic SCI (T8) during the first week postinjury (Riegger et al. 2007). Another study reported a strong increase in the hormones secreted by the adrenal glands (corticosterone and epinephrine) accompanied by an alteration of the synthesis of antibodies (IgG1) in mice with T3 SCI lesions 24 h postinjury, with a return to normal after 3 days (Lucin et al. 2007). Others have reported that the level of SCI has no effect on macrophages and lymphocytes since these cells decreased in both patients with cervical and thoracic SCI (Cruse et al. 2000; Riegger et al. 2007). The findings are conflicting and some studies report that the circulating immune cells increase (Bao et al. 2009) or alternatively decrease in the blood of cervical and thoracic patients (Campagnolo et al. 2000; Cruse et al. 2000). Our results are in conflict with those reported by Gris et al. (2008) since we found that the level of SCI has no impact on pulmonary neutrophils count. Nevertheless, in view of the disparity of results obtained in the clinical context of post‐SCI immune response and the lack of data regarding the lungs, our results offer a first step toward the understanding of the SCI effect on the pulmonary inflammatory response under MV.

As far as we know, immune system function abnormalities may be the cause of diseases by immune deficiency or inappropriate immune responses. Important links between the great body control systems such as the central nervous system (CNS) and the immune system have been established (Catania et al. 2009). The CNS receptors perform the integration of information coming from the periphery and related to the state of the immune system mainly controlled by the vagus nerve (Pavlov et al. 2003; Vinik 2012). Also, SCI may decrease immune cells in the blood reflecting immunosuppression, but the effect of the level of SCI on the lungs remains unknown. High SCI that interrupts sympathetic innervation to major immune organs including vagus nerve dysfunction can significantly decrease immune response in the lungs as it is itself closely linked to the extensive territory of the CNS (T5–T9 innervation). New evidence suggests that the inflammation may vary depending on the inhibition or activation of the nerve (Brommer et al. 2016). Our results mainly illustrate this new finding that SCI‐decreased inflammatory responses in the lungs may be related to the immune system inhibition.

To examine the effect of SCI on lung oxidative stress responses, we measured levels of 8‐isoprostane in the BAL fluids of the lung. We found that MV modulates the oxidative response by greatly increasing basal 8‐isoprostane levels in all MV animals compared to NV animals. Since MV alone is well‐known to induce 8‐isoprostane production, this validates our in vivo model. Intracellular HO‐1 levels in total BAL cells were determined as a marker of antioxidative response. HO‐1 is one of the three forms of heme oxygenase expressed in various cell types (Mege et al. 2011; Murray and Wynn 2011). Previous experimental studies with a model of ventilator‐induced lung injury have highlighted the role of HO‐1 in lung injury by showing that the administration of this enzyme reduces the level of proinflammatory cytokines such as TNF‐*α* and IL‐8, but upregulates levels of the anti‐inflammatory cytokine IL‐10 (An et al. 2011). Our findings suggest that SCI may be a positive promoter of lung defense given that our study showed that TL is able to reduce levels of 8‐isoprostane while increasing HO‐1 levels to baseline in NV‐NL animals. In addition, we found that both TL and CL triggered HO‐1 upregulation in ventilated animals, strongly suggesting an attempt to restore an antioxidative state in lungs of ventilated animals by decreasing oxidative burst stress and promoting antioxidative responses. This new concept is supported by our results which show that SCI in NV and MV groups reduced levels of IL‐1*β* while increasing HO‐1 levels. This is also supported by a recent study indicating that IL‐1*β* downregulates HO‐1 levels, while the induction of HO‐1 expression reverses the effects of IL‐1*β* (Clerigues et al. 2013).

Our study has several limitations, including the absence of measured circulating mediators due to inadequate blood samples: our model was first designed to perform blood samples at different time points (2, 4, 8, 10, and 24 h postsurgery) with the objective to analyze the systemic inflammation. Unfortunately, we met irremediable difficulties such as very low blood samples or impossible to collect. Other limitations are the absence of a NV‐CL control group, measurement of cytokine expression without synthesis measures, a restrictive animal physiology model, and the duration of mechanical ventilation (24 h). Also, we did not measure additional lung inflammation like wet/dry ratio or BAL protein contents in our model which could have supplemented our results, but we feel that given the subtle inflammation in our model, impact of such limitations is very low.

Our study sets the first milestone in the exploration of the impact of SCI on the inflammatory processes in the lung. One hypothesis could be that the local inflammatory response at the SCI affects the lung directly, through the nervous system, or indirectly through systemic mediators. Another possibility is that cervical SCI, requiring MV support, may also affect the lung through modification of mechanics or innervation loss. Further studies are warranted to answer these questions.

## Conclusion

In summary, our study provides evidence of an important interaction between inflammatory response induced by MV and SCI. We showed that the mechanical ventilation‐induced pulmonary inflammation is modulated by SCI, particularly by reducing the levels of inflammatory markers and oxidative stress. We demonstrate, for the first time, that SCI, particularly TL, promotes antioxidant responses in the airways, opposing the oxidative stress response induced by MV. Our results have clinical implications because they strongly suggest that effective treatment of ventilated patients with SCI will depend on the severity of the biotrauma.

## Conflict of Interest

None declared.
